# RNA Interference Silences Genes at Post-Transcriptional Level Without Impacting Nascent RNA in Soybean Hairy Roots

**DOI:** 10.3390/plants15121810

**Published:** 2026-06-12

**Authors:** Li Jiang, Jinfeng Pang, Qianyue Bai, Jian Wei, Zhicheng Dong

**Affiliations:** 1 Guangzhou Key Laboratory of Crop Gene Editing, Guangdong Key Laboratory of Plant Adaptation and Molecular Design, Innovative Center of Molecular Genetics and Evolution, School of Life Sciences, Guangzhou University, Guangzhou 510006, China; lijiangres@163.com (L.J.); stardustherb@outlook.com (J.P.); 2 Chinese Medicine Guangdong Laboratory, Zhuhai 519031, China; 3 School of Agriculture, Jilin Agricultural University, Changchun 130118, China; 14704377775@163.com

**Keywords:** soybean, RNA interference, nascent RNA, hairy root system

## Abstract

RNA interference (RNAi) is an effective mechanism for reducing gene expression, therefore enabling the exploration of gene function. Its application in hairy root transient transformation facilitates rapid preliminary evaluation of gene function, specifically tackling the difficulties linked to steady transformation in soybean. The mechanisms of RNAi have been thoroughly investigated in model plants. Nonetheless, it remains ambiguous whether silencing proceeds at the transcriptional or post-transcriptional level in hairy roots. We analyzed RNA levels and phenotype by RNAi targeting four genes (*GmIAA14*, *GmCPC*, *GmPHT1*, and *GmTTG1*) in soybean roots. A reduction in mRNA levels is generally associated with an altered lateral root phenotype. However, the analysis of chromatin-bound/nascent RNAs during the identical RNAi events revealed no substantial decrease in their abundance. Moreover, the functional impairment of *GmDCL2* or *GmDCL3* did not influence RNA interference, indicating either redundancy or the presence of alternate routes for short RNA-mediated RNA interference in soybean hairy roots. Collectively, our findings underscore possible issues associated with the use of the hairy root transformation method for gene transcription studies. The lack of investigation into RNAi effects at the nascent RNA level, even in model plants, requires a re-evaluation of the current knowledge of RNAi mechanisms.

## 1. Introduction

RNA silencing, also known as RNA interference (RNAi), is a process in eukaryotes that downregulates gene expression by post-transcriptional gene silencing (PTGS) or transcriptional gene silencing (TGS) [1]. This process was originally recognized as a component of the eukaryotic defense system against viruses, wherein double-stranded RNAs (dsRNAs) generated by bidirectional transcription suppress pathogen mRNA levels [2,3]. Mature dsRNAs in plants are thought to be generated via several pathways, including hairpin RNAs transcribed from inverted repeat sequence sites, positive-sense transcripts paired with natural antisense transcripts, and dsRNA synthesized by RNA-dependent RNA polymerases (RdRPs) at repetitive sequence sites [4]. Transcription mediated by DNA-dependent RNA polymerase II or IV produces completely complementary dsRNAs, which are then processed into 21–24-nucleotide (nt) short interfering RNA (siRNA) duplexes by type III ribonucleases, specifically Dicer proteins [5]. Plants include four Dicer isoforms (Dicer-like, DCL1–4), with DCL2, DCL3, and DCL4 generating siRNAs of various lengths (22 nt, 24 nt, and 21 nt, respectively) during RNAi [6]. Subsequently, these siRNAs are incorporated into Argonaute (AGO) family proteins to form RNA-induced silencing complex (RISC), which facilitates gene silencing at either the post-transcriptional or transcriptional stage [7].

Unlike PTGS, which promotes mRNA degradation and translational repression, TGS is mostly accomplished through de novo methylation of target loci directed by siRNAs in plants, a process known as RNA-directed DNA methylation (RdDM) [8]. In *Arabidopsis*, Pol IV or Pol II transcribes certain genomic areas to produce single-stranded RNA, which is then turned into dsRNA by the RDR2 protein. Subsequently, dsRNA is processed by the DCL3 protein to generate 24 nt RNA, which is then loaded on the AGO4 protein to form RISC. Furthermore, a plant-specific RNA polymerase, Pol V, transcribes the corresponding areas to generate RNA that serves as a scaffold, interacting with RISC [9]. This complex specifically targets homologous DNA regions and engages the methyltransferase DOMAINS REARRANGED METHYLTRANSFERASE 2 (DRM2) to facilitate de novo DNA methylation, resulting in TGS [10,11].

By employing the endogenous RNAi system, a specific gene can be silenced through the artificial expression of a hairpin RNA that produces siRNA targeting the gene area. This RNAi approach has been shown to effectively reduce mRNA levels across several species and has been extensively applied to investigate gene function [12]. RNAi has been performed on plants via stable or transient transformation to facilitate gene functional investigations. Although data, including elevated DNA methylation levels and reduced mRNA levels linked to siRNAs, suggests both TGS and PTGS effects mediated by RNAi [13,14], direct evidence of RNAi’s influence on transcription is still absent. Nascent RNAs are RNA molecules that are in the process of transcription but remain incomplete [15]. Recently, techniques for identifying nascent RNAs were developed for plant study [16,17,18,19,20,21,22]. In contrast to mature mRNAs, which arise from RNA biosynthesis and degradation processes, nascent RNAs directly indicate transcriptional activity [23,24]. Despite the prevalent assumption that RNA interference functions at both transcriptional and post-transcriptional stages, direct evidence from nascent RNA measurements corroborating transcriptional gene silencing in plants is limited. To address this deficiency, we aimed to examine if RNAi may influence nascent RNA levels in soybean hairy roots, using nascent RNA, the intermediate product of transcription, instead of mature mRNA that has undergone post-transcriptional modifications.

We selected four target genes—*GmIAA14*, *GmCPC*, *GmPHT1*, and *GmTTG1*—that are actively expressed in soybean roots. Their changed expression yields measurable root phenotypes, rendering them ideal for linking molecular silencing with observable developmental results. *IAA14* is a member of the Aux/IAA family, which is rapidly induced by auxin and acts as a repressor of auxin-responsive transcription, therefore regulating plant growth and development [25]. Comprehensive research from *Arabidopsis* has shown that modified *IAA14* expression substantially influences lateral root development [26]; likewise, the suppression of its soybean homologous gene *GmIAA14* results in a significant decrease in lateral roots, yielding a clear and easily quantifiable phenotype for assessing RNAi effectiveness. The Phosphate Transporter 1 (PHT1) family is essential for processes dependent on inorganic phosphate (Pi). In soybean, *GmPHT1* is upregulated in roots under low external phosphate conditions and operates as a high-affinity phosphate transporter; its root-specific expression pattern enables the evaluation of RNAi effects within a specific tissue context [27]. *TTG1* encodes a WD40 repeat protein crucial for root hair patterning; loss-of-function mutations disrupt root epidermal cell differentiation and result in a significant reduction in root hairs [28]. The MYB transcription factor CPC regulates the transfer of Pi from non-hair cells to hair cells, hence affecting root hair formation [29]; in conjunction with *TTG1*, these two genes offer complementary insights into root-hair-related abnormalities. By focusing on these functionally well-characterized genes, we sought to evaluate RNAi effects on nascent transcripts against a background of well-defined and easily quantifiable root abnormalities, facilitating a clear differentiation between transcriptional and post-transcriptional silencing results.

## 2. Results

### 2.1. RNAi Downregulates the mRNA of Target Genes

**Figure 1:**
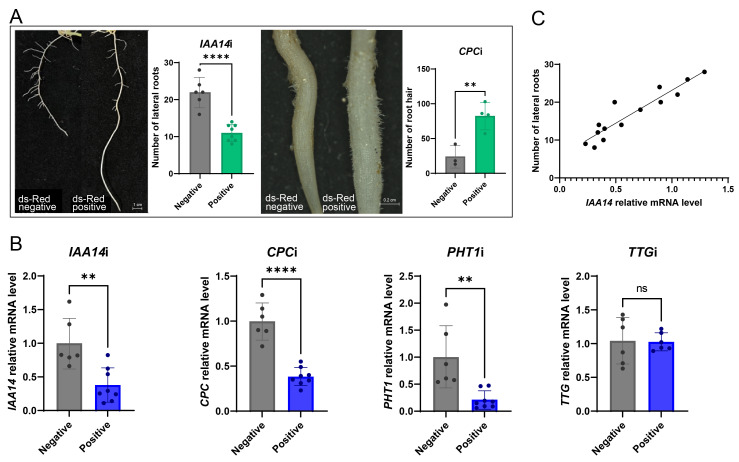
The impact of RNAi on phenotype and mRNA level in soybean hairy roots. (**A**) The number of lateral roots in *GmIAA14*i and root hair in *GmCPC*i RNAi transgenic lines. The transgenic roots were chosen by the red fluorescent marker as dsRed-positive lines. The number of lateral roots was observed under a stereomicroscope and is shown in the bar charts. Values are shown as mean ± SD (n = 3~8). (**B**) Expression of RNAi target genes in the transgenic roots determined by RT-qPCR from total RNA. The relative expression levels were normalized to *GmTubulin* (Glyma.05G157300). Values are shown as mean ± SD (n = 6~8). The *p* value between dsRed-positive lines and dsRed-negative lines was determined by employing the Student’s *t*-test. **** *p* value < 0.0001. ** *p* value < 0.01. ns: not significant. The dots represent individual data points. (**C**) Correlation analysis between the number of lateral roots and the relative mRNA levels of *GmIAA14* in RNAi transgenic lines.

Expression cassettes for the inverted stem–loop hairpin producing short RNA that targets the specified gene (Table S1), along with an *AtUBQ10*-driven dsRed serving as a transgenic positive selection reporter, were integrated into a binary vector named pGD02 (Figure S1). The soybean roots were co-cultured with *Agrobacterium* harboring the binary vector and subsequently selected under fluorescent protein lamp for positive transformation (Figure S2). Compared to the non-transgenic root (dsRed-negative), the number of lateral roots in *GmIAA14*i roots (dsRed-positive) was significantly reduced. The roots of *GmCPC*i showed a considerable increase in root hair production (Figure 1A).

Furthermore, we quantified the mRNA levels of the four genes using quantitative real-time PCR (RT-qPCR). The results demonstrated that the mRNAs of *GmIAA14, GmCPC*, and *GmPHT1* were effectively downregulated by 62.14%, 61.69%, 78.74%, respectively (Table S2). Regarding *GmTTG1*i, we were unable to detect a significant reduction in mRNA levels in the transgenic positive roots. For *GmPHT1*i, despite the proven successful knockdown of the target mRNA, we did not see any notable phenotypic changes in the roots. This may indicate functional redundancy or compensation mechanisms, which is not surprising for a phosphate transporter.

Moreover, a positive correlation (R^2^ = 0.87, r = 0.93, simple linear regression R squared and Pearson correlation) was observed between the relative mRNA level of *GmIAA14* and the associated number of lateral roots (Figure 1B,C and Table S3). The high correlation coefficient indicates that the degree of target mRNA knockdown closely aligns with the severity of the lateral root phenotype, where more effective silencing results in a proportionally greater reduction in lateral root number. This quantitative relationship confirms that RNAi, given through the soybean hairy root transformation system, efficiently inhibits gene expression at the mRNA level to a degree that yields biologically significant developmental outcomes. The strong correlation between molecular silencing and phenotypic expression substantiates the application of this experimental paradigm for investigating RNAi mechanisms, establishing a robust basis for the following comparison of nascent RNA and mature mRNA levels under functionally effective RNAi settings.

### 2.2. RNAi Downregulates the mRNA of Homologous Genes

**Figure 2:**
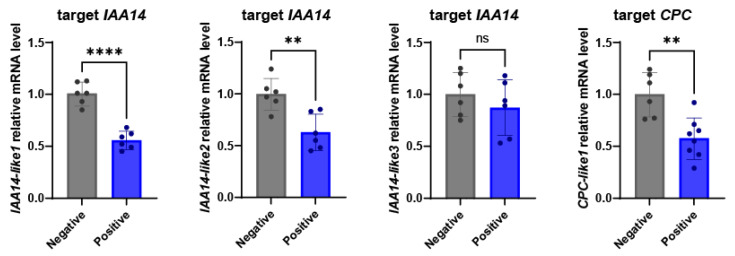
The mRNA level of orthologous genes in transgenic RNAi roots. Orthologous gene expression levels in the transgenic *IAA14* RNAi roots and *CPC* RNAi roots were determined by RT-qPCR from total RNA. The relative expression levels were normalized to *GmTubulin*. Values are shown as mean ± SD (n = 6~8). The *p* value between dsRed-positive lines and dsRed-negative lines was determined by employing the Student’s *t*-test. **** *p* value < 0.0001. ** *p* value < 0.01. ns: not significant. The dots represent individual data points.

Soybean experienced two whole genome duplications, leading to the emergence of functionally redundant gene copies. We identified the three closest homologs of *GmIAA14* (*GmIAA14-like1*, *GmIAA14-like2*, and *GmIAA14-like3*, exhibiting protein sequence similarities of 91.6%, 73.5%, and 73.4% and nucleotide sequence similarities of 90.5%, 58.8%, 57.0%, respectively) and one homolog of *GmCPC* (*GmCPC-like1*, showing 96.2% protein sequence similarity and 83.8% transcript sequence similarity) to evaluate the knockdown efficiency on the homologous genes of target genes. RT-qPCR analysis revealed that, in the *GmIAA14*i transgenic roots, the mRNA levels of *GmIAA14-like1* and *GmIAA14-like2* were slightly downregulated (by 44.63% for *GmIAA14*-like1 and 36.89% for *GmIAA14-like2*), whereas *GmIAA14-like3* exhibited no change. Similarly, *GmCPC-like1* mRNA exhibited a reduction of 42.25% in the *GmCPC*i transgenic roots (Figure 2 and Table S2).

Together, our findings suggest that the RNAi constructs targeting *GmIAA14* and *GmCPC* can effectively cross-silence closely related homologs, with the degree of downregulation corresponding to sequence similarity. The observation that *GmIAA14-like1*, which has the highest nucleotide identity (90.5%) with the target gene, demonstrated the most significant off-target silencing, while the more divergent *GmIAA14-like3* was unaffected, suggests that sequence identity thresholds regulate the degree of homology-dependent silencing. This pattern is consistent with the established mechanism of siRNA-mediated target recognition and underscores the necessity of accounting for potential off-target effects when analyzing RNAi phenotypes, especially in polyploid species like soybean, where functional redundancy among gene family members is common.

### 2.3. Nascent RNA Remains Unchanged During the RNAi Process

**Figure 3:**
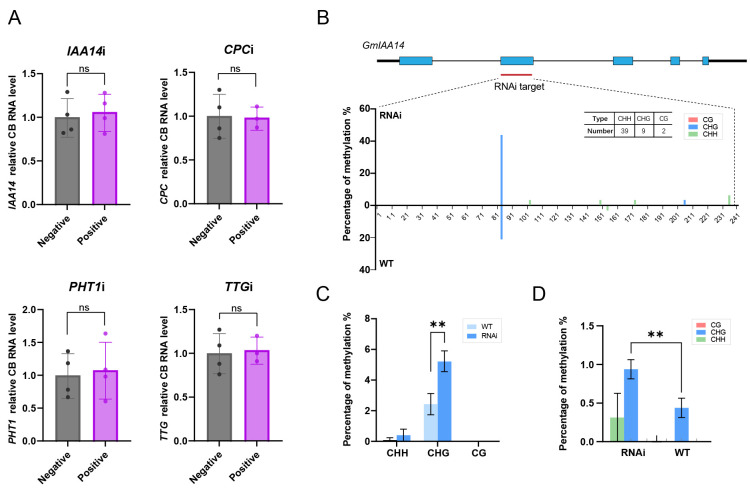
The nascent RNA and target gene methylation level in transgenic RNAi roots. (**A**) The relative RNA expression levels were normalized to *GmTubulin*. Values are shown as mean ± SD (n = 3~4). The *p* value between dsRed-positive lines and dsRed-negative lines was determined by employing the Student’s *t*-test. ns: not significant. The dots represent individual data points. (**B**) DNA methylation level in the target area. The single base in the area is marked to range from 1 to 240 bp. The ratio of clones with methylation detected at each locus to the number of all eight clones shows the methylation percentage at that locus. The table shows the total number of three methylation sites (CG, CHG, and CHH) in the target area. (**C**) Percentage of methylation of different methylation sites in RNAi transgenic roots and wild-type control. Values are shown as mean ± SD (n = 6~8). ** *p* value < 0.01. (**D**) Total methylation level compared to all methylation sites in RNAi transgenic roots and wild-type control ** *p* value < 0.01. Four biological replicates were tested, and eight PCR clones per individual were detected for methylation sequencing. Values are shown as mean ± SD (n = 6~8).

To investigate whether RNAi operates at the transcriptional and/or post-transcriptional level, we examined the chromatin-bound RNA (CB RNA) as an indicator for nascent RNA from the transgenic roots (*GmIAA14*i, *GmCPC*i, *GmPHT1*i, and *GmTTG1*i). The abundance of CB RNAs from these genes were subsequently quantified by RT-qPCR. CB RNAs from the dsRed-positive roots demonstrated a statistically comparable amount to those from the dsRed-negative roots (Figure 3A and Table S2). Consequently, we conclude that neither the transcription rates nor the nascent RNA stability in the hairy roots was altered by the presence of the hairpin RNA.

We subsequently enquired if RNAi modified the level of DNA methylation in this investigation. DNA methylation of the specific region of *GmIAA14* was measured via bisulfite sequencing. The results showed that the methylation level was indeed increased in the RNAi transgenic roots compared to the non-transgenic roots; however, it remained comparatively low in relation to the average methylation level of genes in soybean roots. The target region spans 240 base pairs and contains 50 putative methylation sites, of which only six were confirmed as methylated (Figure 3B and Figure S3). Methylated CHH- and CHG-type sites accounted for only 0.4% and 5% of all potential methylated sites within the target sequences, while CG-type sites exhibited nearly undetectable methylation (Figure 3C). Less than 1% of the cytosine in the overall target region was methylated (Figure 3D). Based on the observed evidence, we speculated that: (1) within the two-week time window of RNAi in soybean hairy root transformation, short RNAs are insufficient to trigger substantial de novo DNA methylation at the targeted locus, and (2) consequently, this minimal enhancement in DNA methylation is insufficient to inhibit transcription.

### 2.4. RNAi Does Not Depend on GmDCL2 or GmDCL3

**Figure 4:**
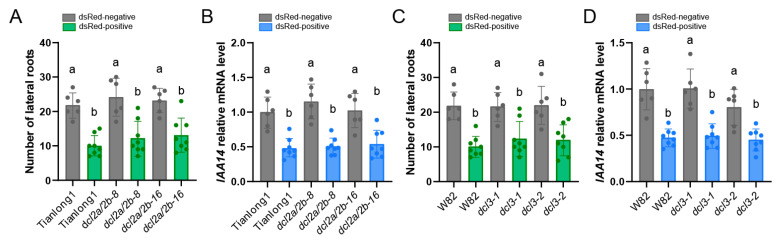
The phenotype of lateral roots and *IAA14* mRNA level of *IAA14*i transgenic roots in *dcl2a/2b* and *dcl3* backgrounds. (**A**) The number of lateral roots was compared between *IAA14*i transgenic lines in wild-type background Tianlong1 and *dcl2a/2b* mutant background. (**B**) *IAA14* mRNA level of *IAA14*i transgenic roots in *dcl2a/2b* background. (**C**) The number of lateral roots was compared between *IAA14*i transgenic lines in wild-type background W82 and *dcl3* mutant background. (**D**) *IAA14* mRNA level of *IAA14*i transgenic roots in *dcl3* background. The relative mRNA expression levels were normalized to *GmTubulin*. One-way analysis of variance (ANOVA) comparison assessing the differences between dsRed-positive lines and dsRed-negative lines. Different letters indicate statistically significant differences with *p* value < 0.05. The dots represent individual biological replicates. All values are shown as mean ± SD (n = 6~8).

Two soybean mutants (*dcl2a/dcl2b* and *dcl3a*) generated using EMS mutagenesis were utilized to evaluate their effect on mRNA interference. A significant decrease in 22 nt RNA was noted in the *dcl2a/dcl2b* double mutant in a prior work [30]. We compared phenotype differences in RNAi transgenic root lines targeting *GmIAA14* between the wild-type and *dcl2a/dcl2b* mutant alleles. A significant and comparable reduction in the number of lateral roots was observed in both wild-type (Tianlong 1 background) and *dcl2a/dcl2b* mutant roots among the dsRed-positive lines (indicated in green, Figure 4A). Correspondingly, *GmIAA14* mRNA levels were significantly reduced in these lines compared to dsRed-negative controls (indicated in grey, Figure 4B). In the examination of the *dcl3a* mutant (Williams 82 background), the absence of *GmDCL3a* did not significantly affect the RNAi-mediated reduction in lateral root number or *GmIAA14* mRNA levels (Figure 4C,D and Table S2). There are at least two explanations for these observations: (1) the RNAi mechanism at the post-transcriptional level is more complex in soybean roots than in model plants. This complexity may arise from the collective contributions of various sizes of short RNAs by DCLs that facilitate RNAi, which might possibly be elucidated in several DCL mutants; (2) there are other mechanisms for mRNA knockdown mediated by hairpin RNA.

## 3. Discussion

RNAi is well known for its function in initiating post-transcriptional and transcriptional gene silencing in plants. In this work, we noted a substantial decrease in the mRNAs of the targeted genes, although the CB RNAs were unaffected by hairpin RNA. This indicates that RNAi takes place at the post-transcriptional stage rather than the transcriptional stage.

The research on the TGS effect induced by dsRNA remains relatively limited, especially for nascent RNA and/or Pol II occupancy as criteria, which is even less frequently examined. Ouvrard et al. showed in a study on mammalian cells that siRNAs directed at a chromatin-associated non-coding RNA induce its transcriptional silence. This was accomplished by quantifying the nascent RNA from the target loci using metabolic labeling with a nucleotide analog and conducting ChIP of RNA Pol II. Furthermore, the findings demonstrated that this transcriptional silencing transpired in *cis* [31]. In *Drosophila*, dsRNA was demonstrated, by metabolic labeling and nuclear run-on assays, to degrade nascent RNA transcripts from a target region, therefore inducing premature transcription termination [32]. These results suggested an alternative TGS mechanism that differs from the classical DNA-methylation-mediated TGS, as well as a co-transcriptional silencing mechanism that acts directly targets nascent RNA, independently of DNA methylation changes. This study found no significant downregulation of nascent RNA; however, a notable reduction in mRNA levels was observed. This suggests that, in the transient transformation system of soybean hairy roots, the nuclear RNAi pathway may be less active or exhibit tissue specificity or that the regulation of nascent RNA could depend on various small RNAs or Dicer proteins.

In contrast to the stable genetic transformation of entire plants, the *Agrobacterium*-mediated rooting system serves as a transient transformation method. It provides the benefits of elevated transformation efficiency and reduced temporal costs; nevertheless, it is also marked by significant transgenic event mosaicism and spatio-temporal instability in exogenous gene expression. These intrinsic features may, to a certain degree, constrain its capacity to facilitate prolonged and stable nuclear RNAi effects. The implementation of the RNAi mechanism in the hairy root system may be more susceptible to favoring swift post-transcriptional degradation in the cytoplasm, while regulation at the level of newly synthesized nuclear RNA may necessitate a prolonged duration of action or a specific chromosomal context. The mechanism via which RNA interference regulates nascent RNA is unclear, and it is questionable whether a genome-integrated siRNA expression cassette is essential for this activity. The soybean hairy root system may not offer the steady conditions required for RNAi to properly downregulate nascent RNA levels. Similarly, the potential for siRNAs to induce widespread off-target silencing may be partially constrained by the transient and mosaic nature of the system. Nonetheless, genome-wide off-target effects were not directly assessed in this study, and future studies employing small RNA sequencing or degradome analysis would be valuable to comprehensively evaluate the specificity of the RNAi construct.

It should also be noted that the *Agrobacterium rhizogenes* K599 strain used for hairy root induction transfers its Ri-plasmid-borne *rol* genes into the host genome, which are known to alter the biochemical and hormonal milieu of transformed cells and can influence the expression of numerous plant genes [33,34]. In our study, transgenic negative hairy roots subjected to the same transformation procedure but lacking the RNAi construct (separate roots in the same explant) served as controls, ensuring that both RNAi-positive and control groups share an identical *rol* gene background. Any residual effects of *rol* gene expression are therefore present in both groups and do not confound the comparison of RNAi-mediated silencing. This consideration reinforces our overarching message that the hairy root system, while widely used for functional characterization of genes through silencing, should be used with caution when drawing conclusions in transcriptional gene silencing mechanisms research.

This research additionally performed a genetic examination of the functions of soybean Dicer mutants. The findings indicated that the RNAi effect in hairy roots persisted even in mutants deficient in *GmDCL2* or *GmDCL3*. The results indicate the presence of functionally redundant Dicer members in soybean or that alternate short RNA synthesis routes, outside of the conventional DCL2/DCL3 pathway, exist to mitigate their functional deficiency. This suggests that the intricacy of the small RNA pathway in soybean may exceed initial expectations; more research employing additional mutant combinations or comprehensive genome-wide small RNA sequencing is required to elucidate the functional differentiation among DCL members in RNA interference. In light of the lack of direct data, including measures of nascent RNA abundance, additional research is warranted, specifically comparing nascent RNA levels in both wild-type and RdDM pathway mutants. It should be noted that our analysis was performed within a 7–14-day window, a period sufficient to establish robust mRNA pools and phenotypes. The absence of nascent RNA reduction during this phase suggests that post-transcriptional silencing is the primary mode of action within this timeframe. We cannot exclude the possibility that longer exposure may eventually affect nascent RNA levels or induce DNA methylation, potentially through distinct mechanisms. Time-course experiments beyond two weeks would be valuable to address this in future studies. Future studies incorporating small RNA analyses may nonetheless help refine our understanding of the specific siRNA species involved and their loading efficiency in this transient system. Consequently, it remains an unresolved inquiry in plants whether nascent RNA can be downregulated by RNAi and what particular conditions are essential to effectively initiate TGS.

## 4. Materials and Methods

### 4.1. Plasmids and Plant Materials

The RNAi vector pGD02-target was constructed from pCAMBIA1300 (Figure S1). In detail, a red fluorescent protein (dsRed) expression cassette was included in the vector for the selection of transgenic roots. A constitutive promoter derived from the soybean gene *GmScreamM4* [35] and a 35S terminator were subsequently included in the vector, with an *Asc*I cleavage site inserted between the promoter and terminator. The target area was about 100 to 500 bp for each gene designated for knockdown. The gene sequences of *GmIAA14* (Glyma.10G180100), *GmCPC* (Glyma.01G224900), *GmPHT1* (Glyma.03G162800), *GmTTG1* (Glyma.06G136900), *GmIAA14-like1* (Glyma.20G210400), *GmIAA14-like2* (Glyma.19G161100), *GmIAA14-like3* (Glyma.02G142500), and *GmCPC-like1* (Glyma.01G224900) were sourced from the Phytozome 13 database. An intron fragment was amplified from pK7GWIWG2DII [36], which expressed the loop area of the hairpin structure. The loop–stem fragments were ligated to the pGD02 vector at *Asc*I using the In-Fusion^®^ HD Cloning Kit (TAKARA, Kyoto, Japan) with two reversed segments of the target area.

The wild-type soybean used for hairy root transformation was either of the Tianlong 1 or Williams 82 background. Mutants *dcl2a/2b-8* and *dcl2a/2b-16* (Tianlong 1 background) were obtained from the Institute of Crop Science, Chinese Academy of Agricultural Sciences [27], and *dcl3a-1* and *dcl3a-2* (Williams 82 background) were obtained by EMS mutagenesis from Nanjing Agricultural University [37]. Soybean plants were cultivated in vermiculite within a greenhouse under a 12 h light/12 h dark cycle at 25 °C.

### 4.2. Soybean Root Transformation

The RNAi constructs were introduced into the *Agrobacterium rhizogenes* strain K599 (WEIDI Shanghai, China). A single colony was selected from freshly grown bacteria on a plate and inoculated into liquid LB medium supplemented with kanamycin for overnight incubation at 28 °C. Then, 200 μL of fresh bacterial culture was spread evenly on the solid LB plate supplemented with kanamycin and incubated at 28 °C for 24–36 h. The procedure of infection followed the method described previously [38]. Explants for hairy root transformation were derived from seedlings that were seven days old post-germination. The explants were cultured for 7 to 14 days after infection. Upon reaching a length above 10 cm, the new roots were extracted from the containers and thoroughly cleaned. The transgenic roots were evaluated in a dark environment using a fluorescent protein lamp (LUYOR-3415, Joliet, IL, USA) at excitation wavelengths of 510–540 nm, with untransformed explants serving as the control group. Positive roots exhibited red fluorescence under the 600 nm filter, whereas negative roots did not. The newly generated roots were subsequently utilized as resources for further phenotypic and molecular research.

### 4.3. Total RNA Extraction and Analysis

Total RNA was extracted using TRIzol™ Reagent (CWBIO, Beijing, China) according to the manufacturer’s instructions. Precisely, 1 mL of TRIzol™ Reagent was used for 100 mg of root tissue that had been ground in liquid nitrogen. Real-time qPCR analysis was done using SYBR Green (CISTRO, Guangzhou, China) following total RNA reverse transcription (TAKARA, Beijing, China). The primers used for RT-qPCR analysis are presented in Table S4. All primers were designed to anneal within a single exon and do not span an intron; therefore, they amplify both nascent and mature transcripts with equal efficiency, ensuring consistent amplification efficiency between total RNA and chromatin-bound RNA measurements.

### 4.4. Chromatin-Bound RNA Extraction and Analysis

Soybean nuclei were prepared [39], followed by the extraction of chromatin-bound RNA, as previously described [40]. Two grams of root material were ground with liquid nitrogen to a fine powder and resuspended in Honda buffer, as modified from reference [41]. The mixture was filtered through two layers of Miracloth. After centrifugation, the supernatant was discarded and an equivalent volume of nuclei suspension buffer was added. The pellet was mixed using a pipette tip, then two volumes of washing buffer were added by aspirating and dispensing. The pellet was resuspended in an equal volume of resuspension buffer and washing buffer. The mixture was subjected to centrifugation to eliminate the supernatant. The RNA attached to chromatin was recovered from the nuclear pellet using the TRIzol RNA extraction procedure. The RNA was incubated with TURBO™ DNase (ThermoFisher, Waltham, MA, USA) for 30 min. The reactant was purified with the RNA Clean & Concentrator™-5 kit (ZYMO research) and the RNA was dissolved in RNase-free water. Reverse transcription and RT-qPCR analysis of nascent RNA were performed using the same primer pairs and the same procedure as described for total RNA, in order to maintain identical amplification conditions and allow direct comparison.

### 4.5. DNA Methylation Analysis

DNA was subjected to bisulfite conversion using the EZ DNA Methylation-Gold kit (ZYMO Research, Irvine, CA, USA) according to the manufacturer’s instructions. Subsequently, the converted DNA was amplified using KAPA HiFi HotStart Uracil ReadyMix (Roche, Basel, Switzerland), with primers listed in Table S4. PCR products were then cloned into pMD19 (TAKARA) and sequenced. The CyMATE tool [42] was employed for the analysis of individual DNA sequences. Both the RNAi dsRed-negative and dsRed-positive lines were examined using four independent biological replicates each. For every biological replicate, 8 individual PCR clones were sequenced to determine the C to T conversion. At each cytosine position and for each methylation context (CG, CHG, and CHH), the methylation percentage was first calculated within that replicate based on the 8 clones. The final methylation percentages presented in the study represent the mean of the values obtained from the four biological replicates. The methylated percentage for each cloned sequence was calculated based on the ratio of unconverted cytosines to the total number of cytosines in the sequence.

## 5. Conclusions

This study advocates for a re-evaluation of the RNAi paradigm in plants, highlighting the importance of directly investigating transcriptional activity to accurately comprehend small RNA-mediated silencing mechanisms. A hairpin-type expression vector was developed and functionally validated for the efficient silencing of target genes and their homologs at the mRNA level. A notable decrease in mRNA levels was reported; however, in the same transgenic events, neither was a similar reduction in nascent RNA levels detected nor were the DNA methylation levels at the target sites modified. Moreover, the efficacy of RNA interference remained unaffected in DCL2- and DCL3-deficient genetic contexts, suggesting the existence of functional redundancy or alternate short RNA pathways in soybean. These data indicate that, while traditional RNAi effectively reduces mature mRNA levels, it does not consistently suppress nascent transcription in soybean hairy roots. The decoupling of mRNA and nascent RNA dynamics challenges established notions regarding the impact of RNA interference on transcription, highlighting the necessity for caution in interpreting gene function studies utilizing this system.

## Data Availability

The original contributions presented in the study are included in the article and Supplementary Materials; further inquiries can be directed to the corresponding authors.

## Supplementary Materials

The following supporting information can be downloaded at: https://www.mdpi.com/article/10.3390/plants15121810/s1, Figure S1: The plasmid maps used in the study; Figure S2: Identification of transgenic positive hairy roots based on RFP fluorescence; Figure S3: DNA methylation of *GmIAA*i target region; Table S1: Sequences of RNAi target region; Table S2: Summary of relative expression for the RT-qPCR result; Table S3: The correlation between the relative mRNA level of *GmIAA14* and the corresponding number of lateral roots; Table S4: Specific primers used in this study; Table S5: Percentage of methylation of different methylation sites in RNAi transgenic dsRed-positive root and dsRed-negative control.

## References

[1] Zhao J., Guo H. (2022). RNA silencing: From discovery and elucidation to application and perspectives. J. Integr. Plant Biol..

[2] Fire A., Xu S., Montgomery M., Kostas S., Driver S., Mello C. (1998). Potent and specific genetic interference by double-stranded RNA in *Caenorhabditis elegans*. Nature.

[3] Waterhouse P., Graham M., Wang M. (1998). Virus resistance and gene silencing in plants can be induced by simultaneous expression of sense and antisense RNA. Proc. Natl. Acad. Sci. USA.

[4] Huang K., Wu X., Fang C., Xu Z., Zhang H., Gao J., Zhou C., You L., Gu Z., Mu W. (2021). Pol IV and RDR2: A two-RNA-polymerase machine that produces double-stranded RNA. Science.

[5] Dunoyer P., Himber C., Ruiz-Ferrer V., Alioua A., Voinnet O. (2007). Intra- and intercellular RNA interference in *Arabidopsis thaliana* requires components of the microRNA and heterochromatic silencing pathways. Nat. Genet..

[6] Vaucheret H., Voinnet O. (2024). The plant siRNA landscape. Plant Cell.

[7] Song X., Li Y., Cao X., Qi Y. (2019). MicroRNAs and their regulatory roles in plant–environment interactions. Annu. Rev. Plant Biol..

[8] Law J., Jacobsen S. (2010). Establishing, maintaining and modifying DNA methylation patterns in plants and animals. Nat. Rev. Genet..

[9] Zhang H., Lang Z., Zhu J. (2018). Dynamics and function of DNA methylation in plants. Nat. Rev. Mol. Cell Biol..

[10] Matzke M., Mosher R. (2014). RNA-directed DNA methylation: An epigenetic pathway of increasing complexity. Nat. Rev. Genet..

[11] Harkess A., Bewick A., Lu Z., Fourounjian P., Michael T., Schmitz R., Meyers B. (2024). The unusual predominance of maintenance DNA methylation in *Spirodela polyrhiza*. G3 Genes Genomes Genet..

[12] Waterhouse P., Helliwell C. (2003). Exploring plant genomes by RNA-induced gene silencing. Nat. Rev. Genet..

[13] Dalakouras A., Wassenegger M. (2021). Viroids as a tool to study RNA-directed DNA methylation in plants. Cells.

[14] Haag J., Pikaard C. (2011). Multisubunit RNA polymerases IV and V: Purveyors of non-coding RNA for plant gene silencing. Nat. Rev. Mol. Cell Biol..

[15] Hetzel J., Duttke S., Benner C., Chory J. (2016). Nascent RNA sequencing reveals distinct features in plant transcription. Proc. Natl. Acad. Sci. USA..

[16] Zhu J., Liu M., Liu X., Dong Z. (2018). RNA polymerase II activity revealed by GRO-seq and pNET-seq in *Arabidopsis*. Nat. Plants.

[17] Kindgren P., Ivanov M., Marquardt S. (2020). Native elongation transcript sequencing reveals temperature dependent dynamics of nascent RNAPII transcription in *Arabidopsis*. Nucleic Acids Res..

[18] Li S., Wang Y., Zhao Y., Zhao X., Chen X., Gong Z. (2020). Global co-transcriptional splicing in *Arabidopsis* and the correlation with splicing regulation in mature RNAs. Mol. Plant.

[19] Szabo E., Reichert P., Lehniger M., Ohmer M., de Francisco Amorim M., Gowik U., Schmitz-Linneweber C., Laubinger S. (2020). Metabolic labeling of RNAs uncovers hidden features and dynamics of the *Arabidopsis transcriptome*. Plant Cell.

[20] Zhu D., Mao F., Tian Y., Lin X., Gu L., Gu H., Qu L., Wu Y., Wu Z. (2020). The features and regulation of co-transcriptional splicing in *Arabidopsis*. Mol. Plant.

[21] Mo W., Liu B., Zhang H., Jin X., Lu D., Yu Y., Liu Y., Jia J., Long Y., Deng X. (2021). Landscape of transcription termination in *Arabidopsis* revealed by single-molecule nascent RNA sequencing. Genome Biol..

[22] Xie Y., Chen Y., Li Z., Zhu J., Liu M., Zhang Y., Dong Z. (2022). Enhancer transcription detected in the nascent transcriptomic landscape of bread wheat. Genome Biol..

[23] Liu M., Zhu J., Dong Z. (2021). Immediate transcriptional responses of *Arabidopsis* leaves to heat shock. J. Integr. Plant Biol..

[24] Zhu J., Zhao H., Kong F., Liu B., Liu M., Dong Z. (2021). Cotranscriptional and posttranscriptional features of the transcriptome in soybean shoot apex and leaf. Front. Plant Sci..

[25] Fukaki H., Tameda S., Masuda H., Tasaka M. (2002). Lateral root formation is blocked by a gain-of-function mutation in the SOLITARY-ROOT/IAA14 gene of *Arabidopsis*. Plant J..

[26] Fukaki H., Taniguchi N., Tasaka M. (2006). PICKLE is required for SOLITARY-ROOT/IAA14-mediated repression of ARF7 and ARF19 activity during *Arabidopsis* lateral root initiation. Plant J..

[27] Fan C., Wang X., Hu R., Wang Y., Xiao C., Jiang Y., Zhang X., Zheng C., Fu Y. (2013). The pattern of Phosphate Transporter 1 genes evolutionary divergence in *Glycine max* L.. BMC Plant Biol..

[28] Galway M., Masucci J., Lloyd A., Walbot V., Davis R., Schiefelbein J. (1994). The *TTG* gene is required to specify epidermal cell fate and cell patterning in the *Arabidopsis* root. Dev. Biol..

[29] Idogawa A., Qin D., Tominaga R. (2023). Amino acid substitution in CAPRICE (CPC) protein affects its cell-to-cell movement in the root epidermis of *Arabidopsis thaliana*. J. Plant Biochem. Biotechnol..

[30] Jia J., Ji R., Li Z., Yu Y., Nakano M., Long Y., Feng L., Qin C., Lu D., Zhan J. (2020). Soybean DICER-LIKE2 regulates seed coat color via production of primary 22-nucleotide small interfering RNAs from long inverted repeats. Plant Cell.

[31] Ouvrard J., Muniz L., Nicolas E., Trouche D. (2022). Small interfering RNAs targeting a chromatin-associated RNA induce its transcriptional silencing in human cells. Mol. Cell Biol..

[32] You J., Song Z., Lin J., Jia R., Xia F., Li Z., Huang C. (2021). RNAi-directed knockdown induces nascent transcript degradation and premature transcription termination in the nucleus. Cell Discov..

[33] Maurizio T., Roberto M., Paolo C. (2018). From *A. rhizogenes* RolD to plant P5CS: Exploiting proline to control plant development. Plants.

[34] Shvets D., Berezhneva Z., Musin K., Baimukhametova E., Kuluev B. (2023). *rol* genes of *Agrobacteria*: Possible biological functions. Biol. Bull. Rev..

[35] Zhang N., McHale L., Finer J. (2015). Isolation and characterization of “*GmScream*” promoters that regulate highly expressing soybean (*Glycine max* Merr.) genes. Plant Sci..

[36] Mohammadi-Dehcheshmeh M., Ebrahimie E., Tyerman S., Kaiser B. (2014). A novel method based on combination of semi-in vitro and in vivo conditions in *Agrobacterium rhizogenes*-mediated hairy root transformation of *Glycine* species. Vitr. Cell Dev. Biol. Plant.

[37] Zhang M., Zhang X., Jiang X., Qiu L., Jia G., Wang L., Ye W., Song Q. (2022). ISOYBEAN: A database for the mutational fingerprints of soybean. Plant Biotechnol. J..

[38] Kereszt A., Li D., Indrasumunar A., Nguyen C., Nontachaiyapoom S., Kinkem M., Gresshoff P. (2007). *Agrobacterium rhizogenes*-mediated transformation of soybean to study root biology. Nat. Protoc..

[39] He S., Su J., Dong Z., Liu M., Zhu J. (2024). Preparation and optimization of nuclei from *Arabidopsis* and soybean for RNA research. Plant Physiol. J..

[40] Liu M., Zhu J., Huang H., Chen Y., Dong Z. (2023). Comparative analysis of nascent RNA sequencing methods and their applications in studies of cotranscriptional splicing dynamics. Plant Cell.

[41] Folta K., Kaufman S. (2006). Isolation of *Arabidopsis* nuclei and measurement of gene transcription rates using nuclear run-on assays. Nat. Protoc..

[42] Hetzl J., Foerster A., Raidl G., Scheid O.M. (2007). CyMATE: A new tool for methylation analysis of plant genomic DNA after bisulphite sequencing. Plant J. Cell Mol. Biol..

