# Supplementary figures and images for: RNA Interference Silences Genes at Post-Transcriptional Level Without Impacting Nascent RNA in Soybean Hairy Roots
Source: Plants (Basel). 2026 Jun 12;15(12):1810. doi: 10.3390/plants15121810 (PMC13307295; doi:10.3390/plants15121810)

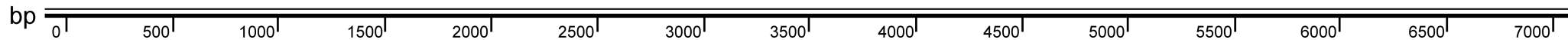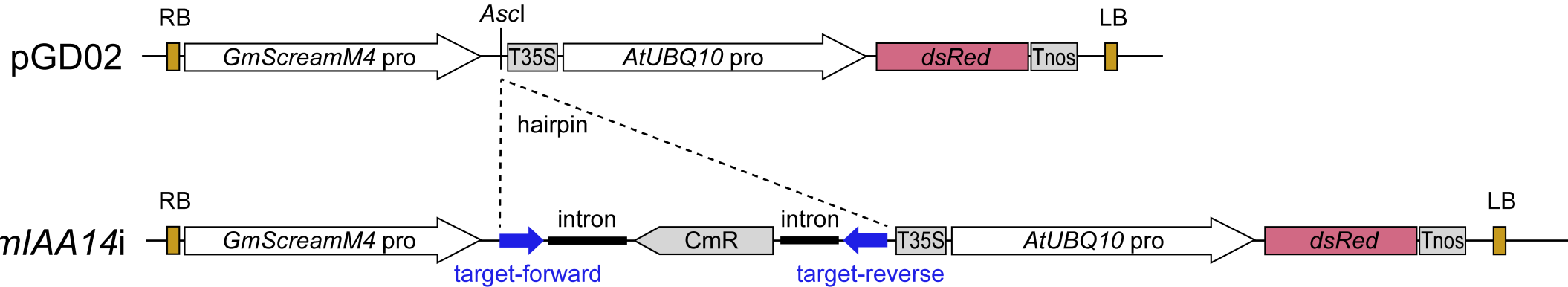

Supplement: Supplementary file 1 [file plants-15-01810-s001.zip › Supplementary Figure S1.pdf]

### DNA methylation of *Gm/AAi* target region

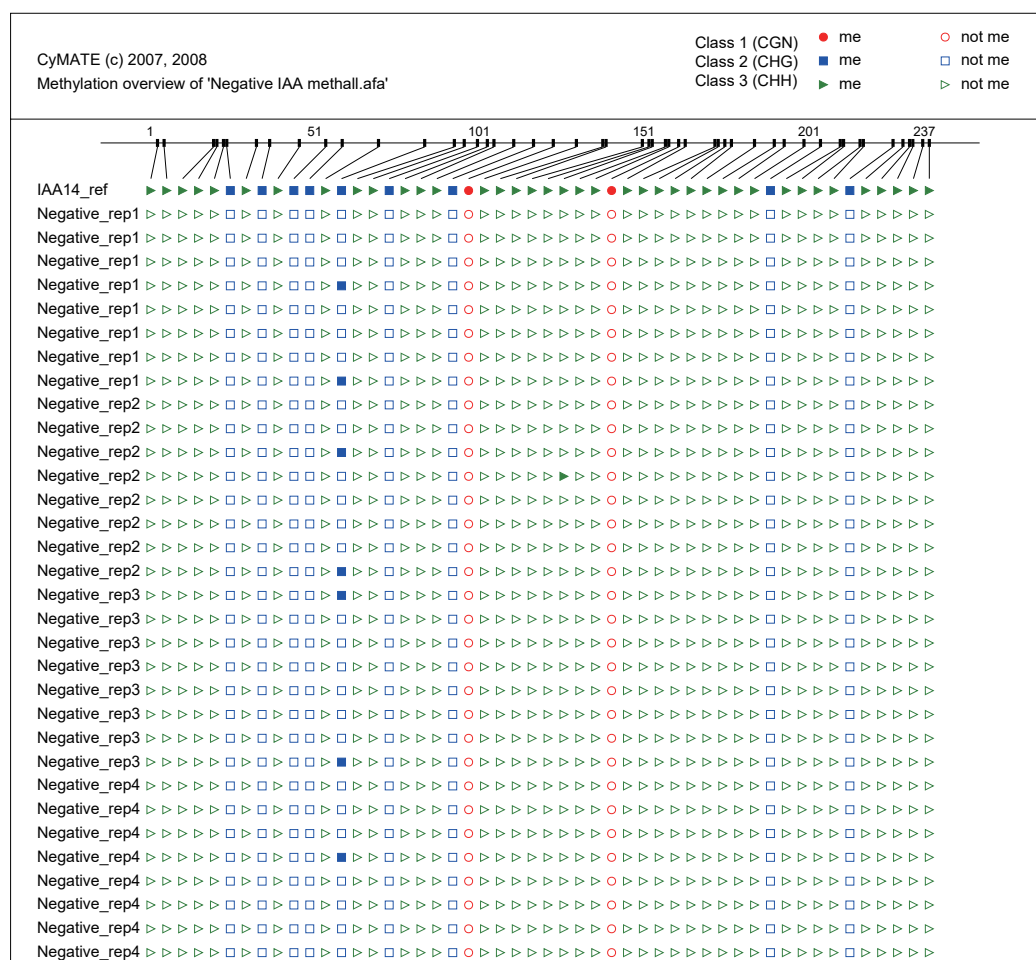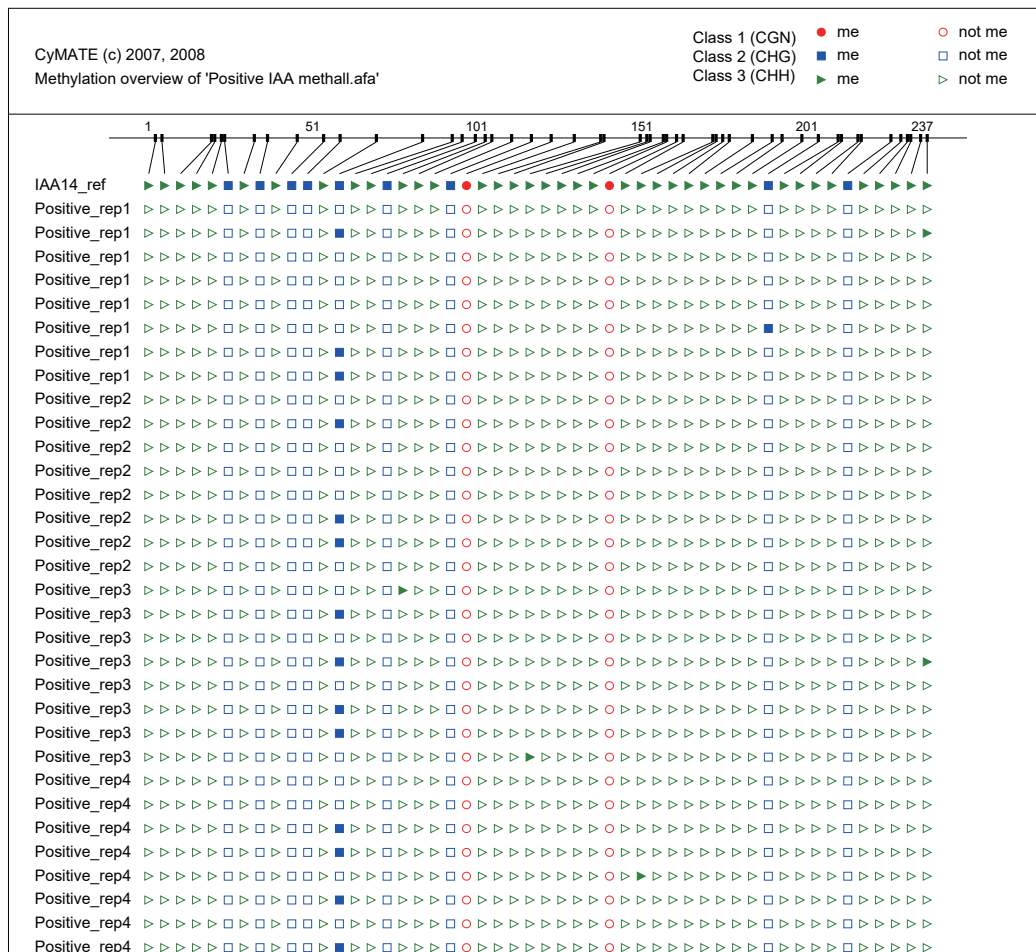

Supplement: Supplementary file 1 [file plants-15-01810-s001.zip › Supplementary Figure S3.pdf]
